# Supplementary material for: Identifying Chemical Differences in Cheddar Cheese Based on Maturity Level and Manufacturer Using Vibrational Spectroscopy and Chemometrics
Source: Molecules. 2023 Dec 12;28(24):8051. doi: 10.3390/molecules28248051 (PMC10745544; doi:10.3390/molecules28248051)
Supplement: Supplementary file 1 [file molecules-28-08051-s001.zip › molecules-2704206-supplementary.pdf]

# Identifying Chemical Differences in Cheddar Cheese Based on Maturity Level and Manufacturer Using Vibrational Spectroscopy and Chemometrics

**Gerson R. Dewantier [1], Peter J. Torley [2] and Ewan W. Blanch [1,*]**

[1] Applied Chemistry and Environmental Science, School of Science, Royal Melbourne Institute of Technology University, Melbourne, VIC 3001, Australia; s3736548@student.rmit.edu.au
[2] Biosciences and Food Technology, School of Science, Royal Melbourne Institute of Technology University, Bundoora, VIC 3083, Australia; peter.torley@rmit.edu.au
* Correspondence: ewan.blanch@rmit.edu.au; Tel.: +61-3-9985-2890

## Supplementary Material

**Table S1.** FTIR band assignment.

| Wavenumber ($cm^{-1}$) | Assignment | Component | Ref. |
|---|---|---|---|
| 3600 – 3000 | O-H str | Water | [1] |
| 3010 | C-H str | Lipids | [2] |
| 2956 | $CH_3$ asym str | Lipids | [3] |
| 2918 | $CH_2$ asym str | Lipids | [3] |
| 2870 | $CH_3$ sym str | Lipids | [3] |
| 2858 | $CH_2$ sym str | Lipids | [3] |
| 1742 | C=O str | Lipids | [3,4] |
| (1690 – 1640) * | $H_2O$ sci | Water | [1] |
| 1636 | $NH_2$ sci amide I | Proteins | [5] |
| 1548 | In plane N-H bend amide II | Proteins | [4] |
| (1516) * | N-H bend | Proteins | [4] |
| 1464 | $CH_2$ sci | Lipids | [4] |
| (1456) * | C-H bend | Lipids | [4] |
| 1414 | =C-H | Unsaturated fat | [6] |
| 1394 | $CH_2$ wagging | Lipids | [4] |
| 1354 | C-H, C-O str | Lipids | [6] |
| 1242 | -C-O str | Lipids, acids | [4] |
| 1174 | C-O ester linkage | Acyls-Glycerol, Lipids | [3] |
| 1098 | -C-O str | Acyls-Glycerol, Fatty acids, Lactic acid | [4] |
| 960 | -HC=CH- (*trans*) | Lipids | [6] |
| 720 | Fat crystals | Lipids | [7] |

*Inside parenthesis: hidden band, or shoulder. Abbreviations: str: stretch; sym: symmetric; asym: asymmetric; sci: scissoring; FAA: Free amino acids.

**Table S2.** Raman band assignment.

| Raman | Assignments | Component | Ref. |
|---|---|---|---|
| 540 | S-S str Cystine | Cysteine | [8] |
| 607 | C-C-O | Lipids | [9] |
| 724 | $CH_2$ | Lipids | [9] |
| 760 | Tryptophan | Tryptophan | [8] |
| 833 | Tyrosine doublet | Tyrosine | [8] |
| 846 | Phosphatidylinositol | Phospholipids | [10] |
| 852 | Phosphatidic acids | Phospholipids | [10] |
| 868 | Phosphatidyl choline | Phospholipids | [11] |
| 889 | Tryptophan | Tryptophan | [8] |
| 924 | C-C | Lipids | [9] |
| 959 | C=C str, C-H def | Unsaturated fatty acids | [9] |
| 990 | C-C str | Lipids | [9] |
| 1004 | Phenylalanine | Phenylalanine | [10,12] |
| 1063 | C-C str | Fatty acids, Lipids | [9] |
| 1080 | C-C str | Saturated fatty acids | [13] |
| 1128 | C-C str | Saturated fatty acids, Lipids | [9] |
| 1158 | β-Carotenoids | Carotenoids | [13] |
| 1266 | =C-H, Amide III | Unsaturated fat, Amide III | [8] |
| 1296 | $CH_2$ twist | Lipids | [9] |
| 1344 | C-H, C-O str | Lipids | [9] |
| 1370 | Tryptophan | Tryptophan | [8] |
| 1421 | =C-H | Unsaturated fatty acids | [9] |
| 1442 | $CH_2$ bend, Amide III | Lipids, Amide III | [13,14] |
| 1526 | Carotene | Carotene | [13] |
| 1554 | N-H, C-N | Protein, Amide II | [9] |
| 1616 | Tyrosine | Tyrosine | [8] |
| 1658 | C=C str, Amide I | Lipids, Amide I | [8] |
| 1742 | C=O | Triacylglycerol ester | [10] |
| 2800-3000 | $CH_2$ | Lipids | [10] |

Abbreviations: str: stretch; def: deformation.

**Table S3.** Composition of the Cheddar cheese samples. *

| | Bega | | | Great Ocean Road | | | |
|---|---|---|---|---|---|---|---|
| | Colby | Tasty | Vintage | Colby | Tasty | Extra-tasty | Vintage |
| Energy (kJ) | 1680 | 1720 | 1720 | 1640 | 1750 | 1750 | 1750 |
| Protein (g/100 g) | 23.3 | 24.2 | 24.8 | 24.5 | 27 | 27 | 27 |
| Fat, total (g/100 g) | 34.4 | 35.3 | 34.7 | 32.5 | 34 | 34 | 34 |
| - Saturated (g/100 g) | 20. 8 | 21.3 | 23.8 | 22.5 | 23.5 | 23.5 | 23.5 |
| Carbohydrate (g/100 g) | < 1 | < 1 | < 1 | < 1 | < 1 | < 1 | < 1 |
| - Sugars (g/100 g) | < 1 | < 1 | < 1 | < 1 | < 1 | < 1 | < 1 |
| Sodium (mg) | 692 | 720 | 650 | 720 | 640 | 640 | 640 |
| Calcium (mg) | 720 | 660 | 738 | 590 | 750 | 750 | 750 |

| | Cracker Barrel | | Mainland | | | | |
|---|---|---|---|---|---|---|---|
| | Vintage | Epicure | Colby | Tasty | Extra-tasty | Vintage | Epicure |
| Energy (kJ) | 1750 | 1750 | 1690 | 1800 | 1800 | 1800 | 1790 |
| Protein (g/100 g) | 25.8 | 25.8 | 23.3 | 23.3 | 23.3 | 23.3 | 22.6 |
| Fat, total (g/100 g) | 34 | 34 | 34.5 | 37.4 | 37.4 | 37.4 | 37.4 |
| - Saturated (g/100 g) | 23.5 | 23.5 | 20.6 | 22.5 | 22.5 | 22.5 | 22.5 |
| Carbohydrate (g/100 g) | < 1 | < 1 | < 1 | < 1 | < 1 | < 1 | < 1 |
| - Sugars (g/100 g) | < 1 | < 1 | < 1 | < 1 | < 1 | < 1 | < 1 |
| Sodium (mg) | 730 | 730 | 674 | 696 | 696 | 696 | 692 |
| Calcium (mg) | 750 | 750 | 650 | 760 | 760 | 760 | 760 |

* From information provided by the manufacturer.

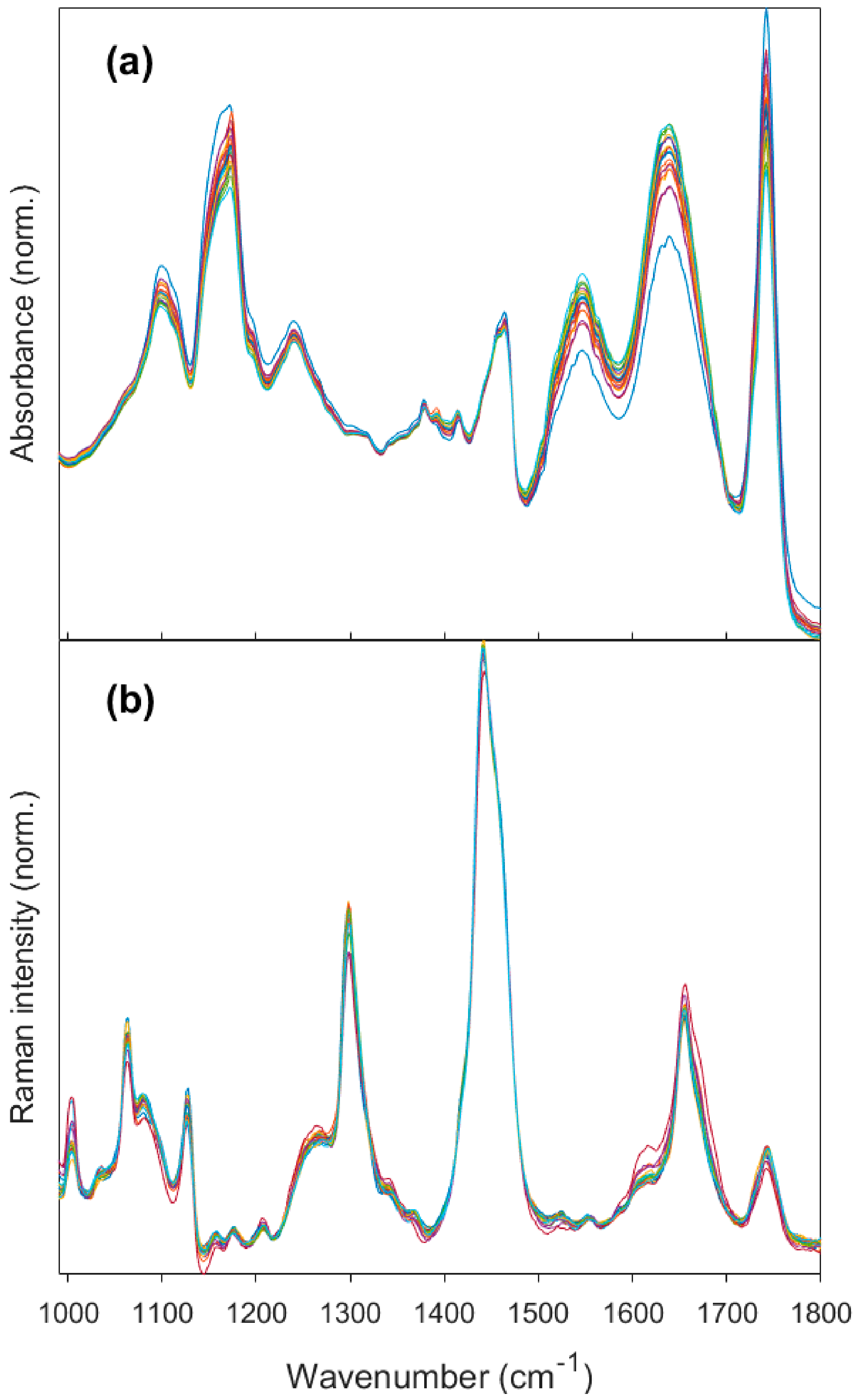


**Figure S1.** Example of variation in spectral data of one of the samples, Bega Colby with a) FTIR spectra and b) Raman spectra.

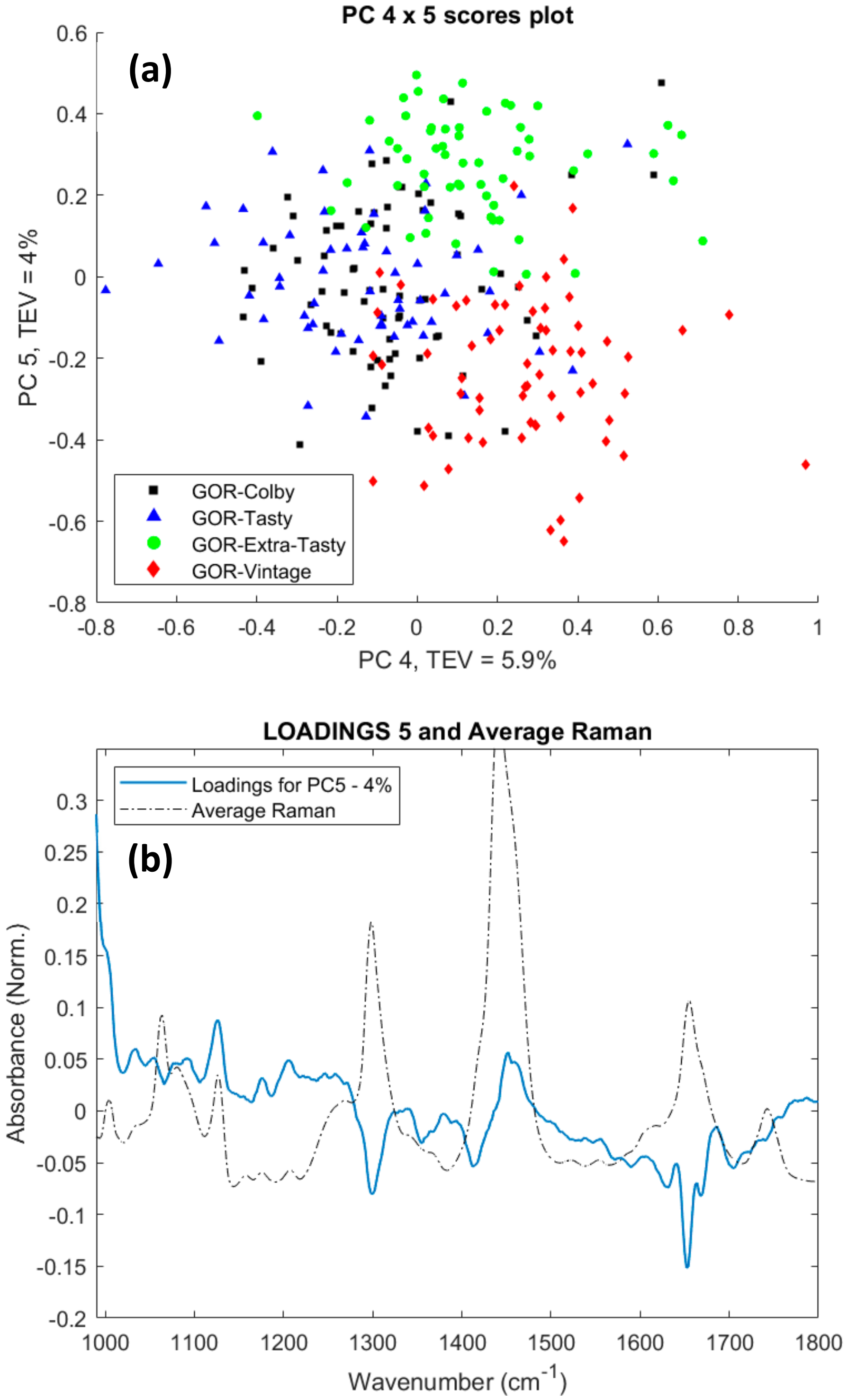


**Figure S2.** a) Scores plot for PCs 4 x 5 of Raman spectra of Great Ocean Road cheeses samples. Note the near total separation between two highest maturity cheeses on PC 5, vertical axis. b) Loadings plot for PC 5 from the PCA of Raman spectra collected on all Cheddar cheese samples.

## References


1. Chen, M.; Irudayaraj, J. Sampling technique for cheese analysis by FTIR spectroscopy. *Journal of Food Science* **1998**, *63*, 96-99.
2. Silverstein, R.M.; Bassler, G.C. Spectrometric identification of organic compounds. *Journal of Chemical Education* **1962**, *39*, 546.
3. Dufour, E.; Mazerolles, G.; Devaux, M.; Duboz, G.; Duployer, M.; Riou, N.M. Phase transition of triglycerides during semi-hard cheese ripening. *International Dairy Journal* **2000**, *10*, 81-93.
4. Karoui, R.; Mouazen, A.M.; Dufour, E.; Pillonel, L.; Schaller, E.; Picque, D.; De Baerdemaeker, J.; Bosset, J.-O. A comparison and joint use of NIR and MIR spectroscopic methods for the determination of some parameters in European Emmental cheese. *European Food Research and Technology* **2006**, *223*, 44-50.
5. Boubellouta, T.; Karoui, R.; Lebecque, A.; Dufour, É. Utilisation of attenuated total reflectance MIR and front-face fluorescence spectroscopies for the identification of Saint-Nectaire cheeses varying by manufacturing conditions. *European food research & technology* **2010**, *231*, 873-882, doi:http://dx.doi.org/10.1007/s00217-010-1336-1.
6. Lerma-García, M.; Gori, A.; Cerretani, L.; Simó-Alfonso, E.; Caboni, M. Classification of Pecorino cheeses produced in Italy according to their ripening time and manufacturing technique using Fourier transform infrared spectroscopy. *Journal of Dairy Science* **2010**, *93*, 4490-4496.
7. Piska, I.; Zárubová, M.; Loužecký, T.; Karami, H.; Filip, V.r. Properties and crystallization of fat blends. *Journal of Food Engineering* **2006**, *77*, 433-438.
8. Li-Chan, E.C. Vibrational spectroscopy applied to the study of milk proteins. *Le Lait* **2007**, *87*, 443-458.
9. Ostovar pour, S.; Afshari, R.; Landry, J.; Pillidge, C.; Gill, H.; Blanch, E. Spatially offset Raman spectroscopy: A convenient and rapid tool to distinguish cheese made with milks from different animal species. *Journal of Raman Spectroscopy* **2021**, *52*, 1705-1711.
10. Gallier, S.; Gordon, K.C.; Jiménez-Flores, R.; Everett, D.W. Composition of bovine milk fat globules by confocal Raman microscopy. *International Dairy Journal* **2011**, *21*, 402-412.
11. Fox, P.; McSweeney, P. Advanced dairy chemistry volume 2 lipids. *New York: Springer* **2006**, *10*.
12. Zhao, M.; Fearon, A.M.; O'Donnell, C.P.; Downey, G.; Beattie, R.J. Prediction of naturally-occurring, industrially-induced and total trans fatty acids in butter, dairy spreads and Cheddar cheese using vibrational spectroscopy and multivariate data analysis. *International Dairy Journal* **2015**, *51*, 41-51, doi:http://dx.doi.org/10.1016/j.idairyj.2015.07.011.
13. Smith, G.P.S.; Holroyd, S.E.; Reid, D.C.W.; Gordon, K.C. Raman imaging processed cheese and its components. *Journal of Raman Spectroscopy* **2017**, *48*, 374-383, doi:http://dx.doi.org/10.1002/jrs.5054.
14. Forrest, G. Raman spectroscopy of the milk globule membrane and triglycerides. *Chemistry and Physics of Lipids* **1978**, *21*, 237-252.
